# Changing burden of cancers related to human papillomavirus in Estonia: a population-based registry study

**DOI:** 10.2340/1651-226X.2026.45671

**Published:** 2026-07-27

**Authors:** Sandra Kase, Mari-Liis Zimmermann, Margit Mägi, Aleksei Baburin, Keiu Paapsi, Reeli Hallik, Piret Veerus, Jana Jaal, Kaire Innos

**Affiliations:** a North-Estonia Medical Center, Oncology Center, Tallinn, Estonia; b National Institute for Health Development, Tallinn, Estonia; c University of Tartu, Institute of Clinical Medicine, Tartu, Estonia

**Keywords:** Human papillomavirus, cancer incidence, epidemiology, vaccination, screening

## Abstract

**Background and purpose:**

Human papillomavirus (HPV) causes multiple cancers. Understanding HPV-related cancer burden may help implement effective strategies for cancer prevention. The aim was to examine incidence and survival trends of eight HPV-related cancer sites in Estonia and estimate the number of cases attributable to HPV.

**Patient/material and methods:**

The Estonian Cancer Registry provided data on all cases of eight HPV-related cancer sites diagnosed in Estonia during 1995–2022. Age-standardized incidence trends were analyzed using joinpoint regression, estimating annual percentage change (APC). The number of HPV-attributable cases was estimated using internationally derived site-specific attributable fractions, as tumor HPV status was not available. Five-year relative survival ratios were calculated from national life tables.

**Results:**

In all, 11,266 cases of HPV-related cancer sites were diagnosed, of which 6,263 were estimated to be attributable to HPV; over 40% occurred before age 55. In women, estimated average annual number of HPV-attributable cases nearly quadrupled for oropharyngeal cancer (OPC) and tripled for anal cancer. A significant increase in OPC and anal cancer incidence was observed among women (APC 10.0 and 3.8, respectively). Cervical cancer incidence declined after 2012 (APC –5.6). Survival improved for OPC in men (from 13 to 44%) and vaginal cancer in women (from 45 to 73%).

**Interpretation:**

HPV-related cancer patterns in Estonia are shifting from cervical to non-cervical cancers. Increasing oropharyngeal and anal cancer incidence highlights the need for prevention strategies beyond cervical screening alone. Strengthening HPV vaccination uptake and sustaining organized cervical screening are critical for reducing future cancer burden.

## Introduction

Human papillomavirus (HPV) is one of the most common sexually transmitted infections worldwide, comprising over 200 genotypes, of which 14 are oncogenic and etiologically linked to a wide spectrum of cancers [1]. Although over 80% of individuals acquire HPV during their lifetime, most infections clear within 1–2 years [2]. However, persistent infection can lead to cancer development. Globally, HPV is responsible for approximately 630,000 new cancer cases annually, accounting for 4.5% of all cancers worldwide [3]. It is estimated that the prevalence of cervical HPV infection, in women with normal cytology, is approximately 12% globally and 14% in Europe [4].

The role of HPV in cervical cancer is well established, yet it is known to contribute to many other types of cancers, such as oropharyngeal, anal, vulvar, vaginal, penile, and, to a lesser extent, laryngeal cancers. There have been suggestions of HPV also having a role in skin and lung cancer development, although the evidence for these sites remains limited [5, 6].

Despite recent gains in cervical cancer control, the incidence of other HPV-related cancers has shown a rising trend. The rise has been particularly marked for oropharyngeal cancer (OPC), increasingly linked to oral HPV infection and changing sexual behaviors [7]. In parallel, HPV-related cancers among men, historically underaddressed, are now a growing public health concern [8].

In Estonia, previous studies have shown a large burden of cervical cancer alongside an increasing incidence of anal and OPC [9–11]. Over the past two decades, Estonia has strengthened cervical cancer prevention through the screening program and the introduction of HPV vaccination into the national immunization program. In 2021, the national cervical cancer screening program transitioned from cytology-based Pap testing to primary HPV testing with partial genotyping. HPV self-sampling in cervical cancer screening was implemented in 2025 to improve participation among under-screened women and is expected to improve both cervical cancer incidence and survival indices. Cervical cancer screening coverage increased from 46.0% in 2015 to 65.2% in 2025 [12].

National HPV vaccination was introduced in Estonia in 2018, initially offering free immunization to girls aged 12–14. Prior to 2018, the HPV vaccines were available but not publicly funded. Since 2024, the national immunization program has become gender-neutral, including all 12–14-year-olds, with a catch-up vaccination up to age 18, with delivery primarily organized through schools. Although the impact of vaccination on cancer incidence is not yet observable within the study time frame, it is warranted to provide a comprehensive assessment of the HPV-related cancer burden in Estonia.

The aim of this study was to examine trends in the incidence and survival of HPV-related cancer sites in Estonia and estimate the number of cases attributable to HPV diagnosed in Estonia, using internationally derived attributable fractions. The findings may provide targeted interventions to reduce disparities and improve outcomes in the population.

## Patients/material and methods

We used data from the Estonian Cancer Registry (ECR), which is a population-based registry with nationwide coverage and data collection since 1968. Reporting to the ECR is mandatory for specialists diagnosing or treating cancer, and case ascertainment is based on multiple sources, including pathology reports, clinical records, linkages with hospital databases of cancer centers, and trace-back of death certificate–initiated cases to health care institutions. The proportion of microscopically verified cases is about 90%, and the proportion of death certificate –only cases is around 2% [13]. The registry is undergoing regular quality control procedures according to international cancer registration standards.

The ECR uses the International Classification of Diseases (ICD)-O-3 for coding tumor topography and morphology and follows international standards and rules issued by the International Association of Cancer Registries and the European Network of Cancer Registries for reporting incidence and survival.

HPV-related cancer sites were defined based on the 10th revision of the ICD-10: oropharynx (codes C01, C02.4, C05.1–2, C09–10), oral cavity (C02–06, excl. C02.4 and C05.1–2), anus (C21), larynx (C32), vulva (C51), vagina (C52), cervix (C53) and penis (C60). Although considered controversial, laryngeal cancer was included based on established international definitions of HPV-related sites, although HPV plays a minor etiological role compared to tobacco and alcohol [3].

The ECR provided data on all incident cases of these sites diagnosed in adults (age ≥15 years) from 1995 to 2022. Only invasive primary malignancies were included; *in situ* lesions were excluded. Multiple primary cancers were counted as separate cases in accordance with international cancer registry rules.

Incidence rates for 1995–2022 were calculated using mid-year population estimates obtained from Statistics Estonia. Age-standardized incidence rates (World Standard Population) were modelled using joinpoint regression to estimate annual percentage change (APC) with 95% confidence intervals (CI).

Joinpoint regression analyses were performed using the Joinpoint Regression Program (Surveillance Research Program, US National Cancer Institute) [14].

For each cancer site, sex, age group, and calendar period, the estimated number of HPV-attributable cancers was obtained by multiplying the observed number of incident cases by the corresponding site-specific attributable fraction reported by de Martel et al. [3], assuming constant fractions over time. Routine tumor-level p16 immunohistochemistry or HPV DNA/mRNA testing was not available for the study period. This approach enables comparability with international burden-of-disease studies, but may underestimate chronological changes, particularly for OPC, where the HPV-attributable fraction has increased in several high-income countries [3]. However, the use of externally derived site-specific attributable fractions is a standard approach in population-based studies in the absence of tumor-level HPV data and allows comparability with international burden estimates.

The following attributable fractions were applied to estimate the number of cancer cases attributable to HPV in Estonia: 50% for oropharynx, 4.3% for oral cavity, 88% for anus, 4.6% for larynx, 24.9% for vulva, 78% for vagina, 100% for cervix uteri, and 51% for penis [3]. To estimate the average annual number of cases, the study period was divided into 4-year intervals, and the total number over each period was divided by four (both for the total number of cases and the estimated number of cases attributable to HPV).

We conducted a preliminary analysis of cases diagnosed in 2020–2022 for which HPV status was reported to the ECR to check whether the applied international estimates are consistent with Estonian data. We included in this analysis sites with at least 10 cases with reported HPV status (cervix, vulva, OPC) and calculated the proportion of HPV-positive cases among all cases with reported HPV status.

Age at diagnosis was categorized as follows: 15–54, 55–64, 65–74, and ≥ 75 years. Due to differences between versions of the classification system and partially incomplete data for earlier periods, tumor stage distribution was assessed for 2018–2022 according to the Union for International Cancer Control (UICC) version 8 of the Tumor, Node, Metastasis(TNM) Classification and grouped into the following categories: I, II, III, IV, and unknown.

For survival analysis, follow-up for vital status from the date of diagnosis until December 31, 2022, was conducted by the ECR at the Estonian Population Registry, using unique personal identification numbers. In case of death or emigration, the respective dates were obtained. Death certificate–only and autopsy cases were excluded from survival analyses. Patients who were diagnosed and died on the same calendar day were included with 1 day of survival time. Relative survival ratio (RSR) was calculated as the ratio of observed survival to expected survival of the underlying general population. The latter was calculated according to the Ederer II method based on national life tables, stratified by age, gender, and calendar year. The study period was divided into 5-year periods: 1998–2002, 2003–2007, 2008–2012, 2013–2017, and 2018–2022. The cohort method was used for patients diagnosed in 1998−2017; the period method for 2018−2022 [15]. RSRs with 95% CIs were calculated using the *strs* algorithm in STATA 18 (StataCorp, College Station, Texas, USA) [16]. International Cancer Survival Standards were used for age standardization [17].

All statistical analyses were performed using Stata 18 (StataCorp, College Station, Texas, USA). Proportions were compared using the chi-square test, with a two-sided *p*-value < 0.05 considered statistically significant. Joinpoint regression analyses were conducted as described above using the Joinpoint Regression Program (Surveillance Research Program, US National Cancer Institute).

The study protocol was approved by the Tallinn Medical Research Ethics Committee.

## Results

### Incidence

**Figure 1:**
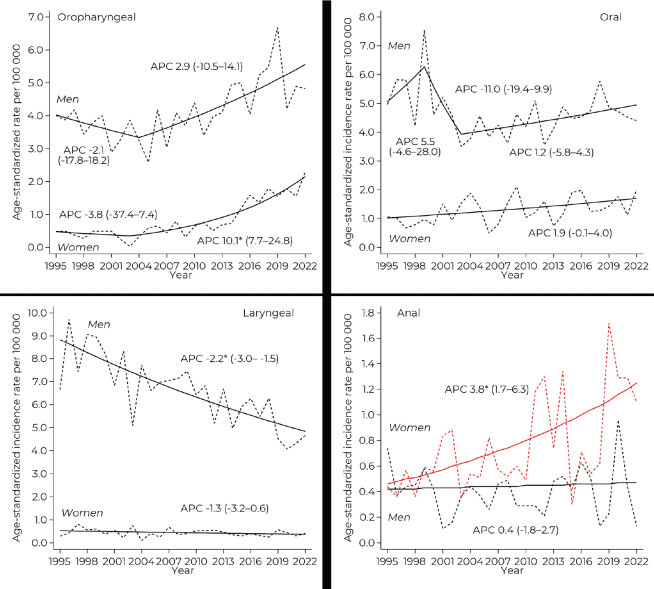
Observed (dotted line) and modelled (solid line) age-standardized incidence trends with annual percentage change (APC) for oropharyngeal, oral, laryngeal, and anal cancer, Estonia, 1995–2022.

**Figure 2:**
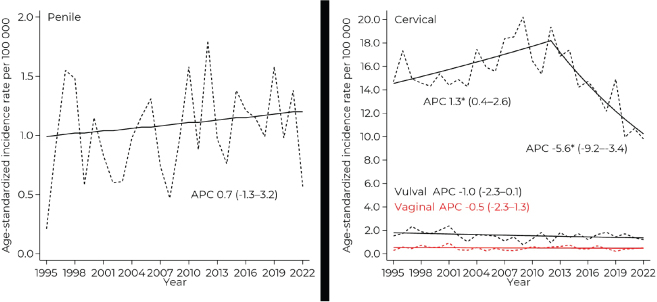
Observed (dotted line) and modelled (solid line) age-standardized incidence trends with annual percentage change (APC) for vulval, vaginal, cervical, and penile cancer, Estonia, 1995–2022.

Figures 1 and 2 present age-standardized incidence trends for cancer sites related to HPV in Estonia during 1995–2022. A rapid increase at a rate of 10.1% per year was seen for OPC in women, driven primarily by the latter part of the study, whereas the 2.9% increase in men was not statistically significant (Figure 1). For oral cavity cancers, the slight upward trend observed was not statistically significant. The decreasing trend for laryngeal cancer is due to the incidence decline seen in men (APC -2.2). The overall increase in anal cancer incidence (APC 2.8) was limited to women (APC 3.8). For vulval and vaginal cancers, there has been a slight non-significant decrease, while a non-significant increase was seen for penile cancer (Figure 2). Cervical cancer incidence increased until 2012 (APC 1.3), followed by a rapid decrease at a rate of 5.6% per year.

**Figure 3:**
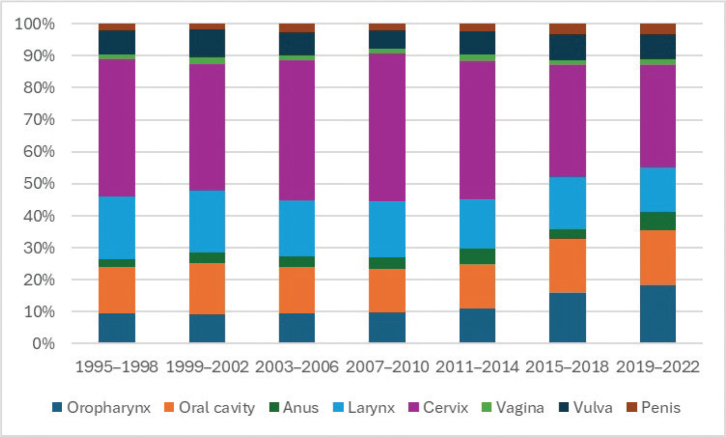
Site distribution of HPV-related cancer sites, Estonia, 1995–2022. HPV: human papillomavirus.

The total number of adult cases of HPV-related cancer sites diagnosed in Estonia during 1995–2022 was 11,266, over 40% of which were cervical, 17% laryngeal, 15% oral cavity, and 12% OPCs (Table 1). From 1995–1998 to 2019–2022, the proportion of cervical cancers decreased from 43 to 32%, while a twofold increase was seen in the proportion of oropharyngeal (from 9 to 18%) and anal cancers (from 3 to 6%) (Figure 3).

**Table 1:** Total number of adult (age ≥15 years) incident cases of HPV-related sites and estimated number of cases attributable to HPV, Estonia, 1995–2022.

Site (ICD-10)	Total no of cases (col%)	HPV-attributable fraction (%)*	Estimated number attributable to HPV						
			Total	Men	Women	Age 15–54	Age 55–64	Age 65–74	Age ≥ 75
Oropharynx (C01, C02.4, C05.1–2, C09–10)	1340 (11.9)	50.0	670	532	139	186	258	168	59
Oral cavity (C02–06, excl. C02.4, C05.1–2)	1716 (15.2)	4.3	74	52	22	18	25	20	11
Anus (C21)	434 (3.9)	88.0	382	98	284	73	90	94	125
Larynx (C32)	1915 (17.0)	4.6	88	81	7	17	31	27	13
Vulva (C51)	837 (7.4)	24.9	208	–	208	17	26	64	101
Vagina (C52)	210 (1.9)	78.0	164	–	164	29	32	44	59
Cervix (C53)	4534 (40.2)	100.0	4534	–	4534	2257	940	774	563
Penis (C60)	280 (2.5)	51.0	143	143	–	31	35	45	32
Total	11,266 (100)	–	6263	904	5358	2628	1437	1237	961

* HPV-attributable fractions from de Martel et al. [3], assuming constant fractions over time. HPV: human papillomavirus; ICD: International Classification of Diseases.

### Estimated number of cases attributable to HPV

Based on internationally derived attributable fractions, the total number of cases attributable to HPV was 6263 (904 in men and 5358 in women) (Table 1). Cervical cancer accounted for 72.4% of the estimated HPV-attributable cancer burden in Estonia.

Men accounted for a larger proportion of HPV-attributable oropharyngeal, oral cavity, and laryngeal cancers, while the opposite was seen for anal cancer. Over 40% were diagnosed at an age below 55 years (20% when excluding cervical cancer).

Among men, the estimated average annual number of HPV-attributable OPC cases nearly doubled over the study period (Supplementary Table 1). In women, the corresponding increase was even more pronounced, with a near fourfold rise in OPC and a threefold rise in anal cancer cases from 1995–2000 to 2019–2022.

In the preliminary analysis of cases diagnosed in Estonia in 2020–2022 for which HPV status was reported to the ECR, the proportion of HPV-positive cases was 96% for cervix, 25% for vulva, 49% for OPC in men, and 66% for OPC in women.

### Survival analysis

**Figure 4:**
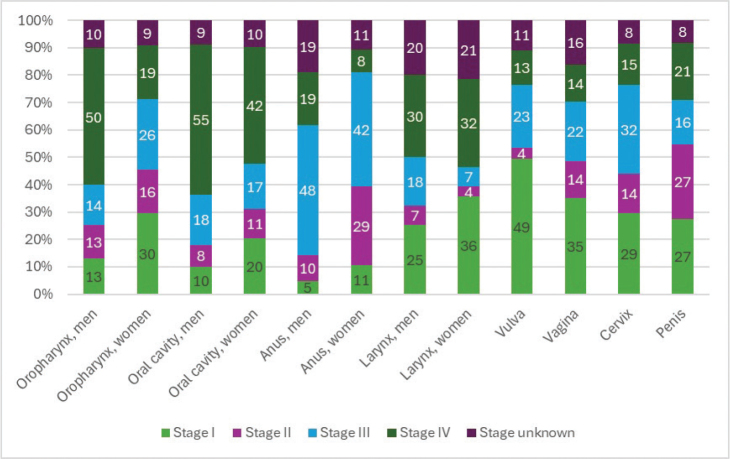
Stage distribution of HPV-related cancer sites, Estonia, 2018–2022. HPV: human papillomavirus.

After the exclusion of death certificate–only (*n* = 76) and autopsy cases (*n* = 60), 11,130 cases were eligible for survival analysis. Overall, 89% of cases had TNM stage reported to the cancer registry (Figure 4). The proportion of unknown stage was below 10% for oropharyngeal, oral, cervical, and penile cancer, but was 20% for laryngeal cancer. Among cases with known stage, the highest proportion of early stage was seen for vulval, vaginal, and laryngeal cancer in women, while the largest proportion of stage IV cancer was seen in oral cavity and OPC in men. For OPC, there was a significant difference in stage distribution between men and women (*p* < 0.001).

Over the study period, age-standardized 5-year relative survival increased the most for OPC in men, reaching 44% (an increase of 31 percentage points) and for vaginal cancer in women, reaching 73% (an increase of 28 percentage points) (Table 2). Survival gains exceeding 20 percentage points were also seen for OPC in women and anal cancer in both men and women. The increase was more modest for penile cancer, vulval and cervical cancer, and laryngeal cancer in men. Survival decreased for laryngeal cancer in women (crude estimates). For OPC and anal cancer, survival increased more among men, resulting in a smaller sex gradient, but the opposite pattern was seen for oral cavity cancer.

**Table 2:** Age-standardized 5-year relative survival ratio (RSR) for HPV-related cancers, Estonia, 1998–2022.

Site	Five-year RSR (95% CI)											
	1998–2002		2003–2007		2008–2012		2013–2017		2018–2022		Change	
	Men	Women	Men	Women	Men	Women	Men	Women	Men	Women	Men	Women
Oropharynx	13 (8–19)	31 (9–57)	13 (8–18)	52 (25–73)	20 (14–25)	25 (13–38)	41 (32–51)	72 (49–86)	44 (34–52)	57 (43–68)	31	26
Oral cavity	20 (13–28)	31 (19–43)	25 (18–32)	45 (34–56)	40 (30–49)	45 (33–55)	44 (34–54)	56 (44–67)	38 (29–47)	55 (43–65)	18	24
Anus	*22 (4–50)*	40 (27–53)	*25 (7–47)*	57 (41–69)	*20 (4–45)*	63 (50–74)	*44 (21–65)*	66 (52–76)	*50 (25–70)*	63 (51–73)	28	23
Larynx	51 (40–60)	*55 (33–72)*	51 (42–60)	*67 (41–83)*	65 (55–73)	*64 (42–80)*	58 (50–66)	*67 (41–84)*	58 (49–66)	*51 (30–68)*	7	-4
Vulva	–	*50 (39–60)*	–	*62 (49–72)*	–	*51 (40–62)*	–	*62 (48–72)*	–	*61 (47–72)*	–	11
Vagina	–	45 (28–60)	–	42 (23–60)	–	56 (37–72)	–	89 (61–97)	–	73 (55–85)	–	28
Cervix	–	60 (56–64)	–	67 (63–71)	–	65 (62–68)	–	67 (63–70)	–	69 (65–72)	–	9
Penis	58 (17–85)	–	71 (44–87)	–	62 (40–78)	–	63 (47–75)	–	71 (49–85)	–	13	–

Estimates in italics are not age-standardized due to the small number. HPV: human papillomavirus.

## Discussion

This population-based study of HPV-related cancer burden in Estonia demonstrated a rapidly increasing incidence of OPC and anal cancer in women, a nonsignificant increase in OPC incidence in men, and a decreasing incidence of cervical cancer in women. These results suggest an epidemiological shift in the HPV-related cancer burden from cervical to noncervical sites, with a multifold increase seen in the average annual number of cases of OPC and anal cancer attributable to HPV. Over the period of 20 years, 5-year relative survival improved considerably for most sites, except for the larynx and cervix. In men, the largest survival gains were observed for OPC and anal cancer; in women, for vaginal cancer and OPC.

The main strength of the study was the availability of high-quality cancer registry data collected uniformly over a long period of time and the ability to show TNM stage distribution for the most recent period.

The main limitation was the absence of long-term routine p16 immunohistochemistry or HPV DNA/mRNA testing in Estonia, which precluded direct identification of HPV-driven tumors at the individual level and necessitated the use of international attributable fraction estimates. These fractions were applied as constants over time, which may have underestimated the HPV-attributable burden in recent years, particularly for OPC, where HPV-attributable fractions have increased in several high-income countries [18]. However, in the absence of historical tumor-level HPV data, this approach represents the most feasible method for long-term population-based analysis and allows comparison with international studies. Also, in the preliminary analysis of cases diagnosed in2020–2022 with reported HPV status, site- and sex-specific HPV-positivity estimates closely aligned with the applied international estimates, except for a higher proportion of HPV-positivity in OPC in women (66% in women vs 50% in men). The latter may have caused underestimation of cases attributable to HPV in women in recent years. Given Estonia’s small population, the number of cases for several sites was limited and susceptible to random fluctuations. To mitigate this, we calculated average annual numbers over a 4-year period. The observed trends may also have been to some extent influenced by changes in diagnostic practices and coding.

Overall, cancer cases attributable to HPV accounted for approximately 3% of all cancers diagnosed in Estonia during the study period. This is slightly lower than the worldwide estimate of 4.5%, but higher than the European estimate of 2.5% [3]. Cervical cancer still accounts for a majority of all HPV-attributable cancer cases, 72.4%, compared to 67% in Europe [3].

Between 1995 and 2022, the most dominant increase was the 10% annual increase observed for OPC in women. This rapid rise is comparable to that of many high-income countries. In the US, the incidence of HPV-positive OPC increased by ~225% from 1988 to 2004, averaging ~8–9% per year [19]. Population-based data from Northern Europe have demonstrated a marked increase in HPV-related OPC over recent decades [18, 20].

The increase in OPC incidence in men was not statistically significant, possibly due to the already high baseline at the beginning of the study period, driven by risk factors other than HPV, such as tobacco and alcohol use, which may have partially masked HPV-related increases. At the beginning of the 2000s, when the current increase started, the incidence of OPC in men was 10 times higher than in women, but the difference declined to 2.5 times by the end of the study period. Our preliminary analysis also showed a higher proportion of HPV-positive OPC cases in women than in men, further supporting the hypothesis that the rapid increase in women is likely driven by HPV burden. This is consistent with evidence suggesting shared HPV-related risk patterns between cervical and OPC in women [21].

The overall increase in anal cancer incidence was limited to women. The finding is consistent with the global rise of anal cancer incidence and with data from the Nordic countries [22, 23]. Data from the US showed an annual incidence increase of 2.7% between 2001 and 2015, accompanied by a 3.1% annual rise in mortality [24]. Global rise is thought to be linked to the high prevalence of persistent HPV infection and to sexual behaviors, including the number of sexual partners and receptive anal intercourse, lack of screening, and low HPV vaccination [25].

The higher and more rapidly increasing incidence of anal cancer among women may partly reflect the strong association between anal cancer and prior HPV-related anogenital disease, including cervical intraepithelial neoplasia and cervical cancer. Women with a history of cervical dysplasia or cervical cancer have been shown to carry an increased long-term risk of anal cancer [26]. The rapid rise in anal cancers in women in Estonia is consistent with earlier evidence of increasing risk of cervical cancer in successive birth cohorts born after 1940 and supports HPV as the central etiologic factor [11].

Cervical cancer incidence has been in a sustained and statistically significant decline starting from 2012, which likely reflects the cumulative impact of the organized screening program introduced in 2006 and increasing screening coverage over time [27].

However, the persistently high number of cases suggests high prevalence of HPV in the population [28], suboptimal screening participation, especially in more vulnerable subgroups and those without health insurance [11], and suboptimal quality assurance and follow-up in the screening program [29, 30]. The transition from cytology-based screening to primary HPV testing and inclusion of uninsured populations in 2021 occurred late in the study period and is therefore unlikely to have substantially influenced the incidence trends observed up to 2022.

Oral cavity and laryngeal cancers showed stable or declining trends in Estonia, largely due to decreasing rates in men. These findings are consistent with previous studies in Estonia and elsewhere [7, 10]. Because HPV has a limited and less certain causal role in oral cavity and laryngeal cancers, the estimated HPV-attributable burden for these sites should be interpreted with particular caution, as trends are more likely to reflect changes in behavioral risk factors rather than HPV epidemiology.

Vulval, vaginal, and penile cancers represent a heterogeneous group. A slight nonsignificant decrease in their incidence possibly reflects an interplay between HPV exposure, screening practices, and sexual behavior patterns, as only a proportion of those cancer diagnoses are related to HPV infection [1].

Our data revealed significant disparities in stage distribution between genders, as men exhibited a higher proportion of stage IV disease for OPC anal oral cavity cancer. The survival gap between men and women also persists, which may partly reflect different care-seeking behavior and treatment compliance but also differences in the proportion of HPV-positive cases, as HPV-positive OPC cases have been shown to have better outcomes [18, 31].

The 5-year relative survival for men in OPC has increased markedly, by 31% over the 20-year study period. The survival improvement was considerably larger for OPC compared to oral cancer, possibly resulting from the rising proportion of HPV-attributable OPC cases. Nevertheless, Estonian survival estimates remain about 20 percentage points or more below those seen for the Nordic countries, particularly in men [32]. The observed lower survival likely suggests a combination of factors. A considerable proportion of patients in Estonia continues to be diagnosed with advanced-stage disease, thus limiting the potential for curative treatment. Delayed diagnosis may also partly reflect lower awareness of early symptoms, delayed healthcare-seeking behavior, and inequalities in access to preventive and diagnostic services. Compared with Nordic countries, multidisciplinary treatment pathways, centralization of complex oncologic care, and access to modern diagnostics and supportive care have evolved later in Estonia. Routine determination of HPV or p16 tumor status has not been systematically implemented in Estonia, which limits risk stratification and prognostic classification. Persistent socioeconomic inequalities, including lower participation in screening and potentially delayed referral in vulnerable populations, may further contribute to poorer outcomes compared with Nordic countries, where preventive programs and healthcare access have historically achieved broader population coverage. For cervical cancer, the limited survival improvement suggests that reductions in case numbers have not yet translated into sufficient improvements in early detection, timely diagnostic follow-up, or treatment outcomes among women who still develop invasive disease. This may indicate persistent barriers among socioeconomically vulnerable and underscreened populations, who remain more likely to present with advanced disease despite improvements in population-level prevention [33].

Compared with Nordic and Western European countries, Estonia demonstrates a similar epidemiological transition, with declining cervical cancer incidence and increasing burden of noncervical HPV-related cancers [1]. However, the relative contribution of cervical cancer remains higher than in many Western and Nordic countries.

While organized cervical screening has been proven effective, no analogous secondary prevention exists for oropharyngeal or anal cancers. Therefore, these findings emphasize the importance of HPV vaccination in both sexes and highlight the need to extend preventive strategies beyond cervical screening alone. Although HPV vaccination in Estonia was introduced for girls in 2018, the uptake has remained suboptimal. In February 2024, Estonia expanded the vaccination program to a gender-neutral strategy (boys and girls aged 12–18) and simplified the schedule to a one-dose regimen [34]. Early data suggest strong uptake among boys: in 2024, 22,572 boys were vaccinated, accounting for 75.4% of all HPV vaccinations delivered that year. According to 2024 data, 47% of children aged 12–14 had received the vaccine (54% of girls and 41% of boys) [35]. However, vaccination stays below optimal cancer-prevention levels recommended by the World Health Organization [36]. Vaccination is organized at schools and is therefore accessible to all children, but studies have shown regional differences and a lack of parental readiness to vaccinate their children, which may exacerbate social inequalities in cancer prevention, particularly as current legislation requires active parental consent [37]. Efforts to increase health literacy among both children and their parents are therefore strongly warranted. Beyond vaccination, targeted strategies are needed to improve participation in cervical screening among socioeconomically disadvantaged and uninsured women and vulnerable groups, ensuring high-quality follow-up of abnormal tests, and sustaining recent innovations such as HPV self-sampling. New approaches are also needed to target groups at high risk for anal cancer, such as women with a history of HPV-related anogenital disorder, people living with HIV, or men who have sex with men.

Future registry-based surveillance would benefit from systematic recording of tumor HPV status, particularly for oropharyngeal, anal, and cervical squamous cell carcinomas. Recent evidence also highlights the emerging clinical utility of circulating HPV DNA as a tumor-derived biomarker across HPV-associated squamous cell carcinomas, while emphasizing the need for coordinated studies with adequate sample sizes and validation [38].

Furthermore, as more than 40% of HPV-attributable cancers were diagnosed before the age of 55 years, the societal impact is substantial, as many of these individuals are of working age, raising families, and contributing actively to the workforce. Beyond mortality, HPV-related cancers therefore carry considerable implications for productivity, fertility, long-term morbidity, and healthcare costs**.**

## Conclusion

Estonia is experiencing a dynamic transition in HPV-related cancer epidemiology. While cervical cancer continues to account for the largest proportion of HPV-attributable cancers, the burden of OPC and anal cancers is increasing.

To diminish future HPV-related cancer burden, it is essential to increase uptake of the gender-neutral HPV vaccination program, which has only recently expanded to include boys. Overall health awareness, recognition of symptoms, and timely access to healthcare services may contribute to earlier diagnosis and better outcomes for HPV-related cancers. Further research is needed to identify suitable strategies for reaching the desired vaccination coverage. In addition, it is vital to sustain investments in cervical cancer screening availability for vulnerable groups and quality assurance, and enhance the awareness programs for public education, keeping in mind other HPV-related cancers.

## Supplementary Material



## Data Availability

The data that support the findings of this study are based on records from the ECR. Access to registry data may be granted upon request and with appropriate approvals from the ECR and relevant ethical review authorities, in accordance with national data protection regulations.
