# Supplementary material for: Changing burden of cancers related to human papillomavirus in Estonia: a population-based registry study
Source: Acta Oncol. 2026 Jul 27;65:45671. doi: 10.2340/1651-226X.2026.45671 (PMC13421172; doi:10.2340/1651-226X.2026.45671)
Supplement: Supplementary file 1 [file AO-65-45671-s1.pdf]

Supplementary material has been published as submitted. It has not been copyedited, or typeset by Acta Oncologica

Supplementary Table 1: Estimated average annual number of cases attributable to HPV by period of diagnosis, Estonia 1995–2022

	Estimated average annual number attributable to HPV													
	1995–1998		1999–2002		2003–2006		2007–2010		2011–2014		2015–2018		2019–2022	
Site (ICD-10)	Men	Women	Men	Women	Men	Women	Men	Women	Men	Women	Men	Women	Men	Women
Oropharynx (C01, C02.4, C05.1–2, C09–10)	32	6	30	6	30	5	35	6	38	9	49	17	53	22
Oral cavity (C02–06, excl. C02.4, C05.1–2)	44	12	48	16	36	19	37	19	42	16	47	23	47	24
Anus (C21)	5	6	3	10	3	10	4	11	4	18	4	9	5	19
Larynx (C32)	71	6	71	5	61	5	66	6	58	7	62	5	50	6
Vulva (C51)	-	29	-	34	-	27	-	24	-	30	-	33	-	33
Vagina (C52)	-	7	-	9	-	6	-	6	-	10	-	7	-	8
Cervix (C53)	-	168	-	155	-	164	-	190	-	183	-	144	-	130
Penis (C60)	9	-	7	-	10	-	9	-	11	-	14	-	13	-
